# How well do you see what you hear? The acuity of visual-to-auditory sensory substitution

**DOI:** 10.3389/fpsyg.2013.00330

**Published:** 2013-06-18

**Authors:** Alastair Haigh, David J. Brown, Peter Meijer, Michael J. Proulx

**Affiliations:** ^1^Crossmodal Cognition Laboratory, School of Biological and Chemical Sciences, Queen Mary University of LondonLondon, UK; ^2^Metamodal BVEindhoven, Netherlands; ^3^Crossmodal Cognition Laboratory, Department of Psychology, University of BathBath, UK

**Keywords:** sensory substitution, blindness, acuity, synthetic synesthesia, visual, auditory

## Abstract

Sensory substitution devices (SSDs) aim to compensate for the loss of a sensory modality, typically vision, by converting information from the lost modality into stimuli in a remaining modality. “The vOICe” is a visual-to-auditory SSD which encodes images taken by a camera worn by the user into “soundscapes” such that experienced users can extract information about their surroundings. Here we investigated how much detail was resolvable during the early induction stages by testing the acuity of blindfolded sighted, naïve vOICe users. Initial performance was well above chance. Participants who took the test twice as a form of minimal training showed a marked improvement on the second test. Acuity was slightly but not significantly impaired when participants wore a camera and judged letter orientations “live”. A positive correlation was found between participants' musical training and their acuity. The relationship between auditory expertise via musical training and the lack of a relationship with visual imagery, suggests that early use of a SSD draws primarily on the mechanisms of the sensory modality being used rather than the one being substituted. If vision is lost, audition represents the sensory channel of highest bandwidth of those remaining. The level of acuity found here, and the fact it was achieved with very little experience in sensory substitution by naïve users is promising.

## Introduction

Do we see with the eyes or with the brain? Is vision a discrete form of perception, distinct from others such as audition and touch? Is it possible for those who have lost their eyesight or have been born without vision to experience visual sensation or perception? Questions such as these have occupied the minds of philosophers and scientists for centuries (Morgan, 1977) and now lie at the heart of modern cognitive neuroscience. Today, with current experimental techniques and technologies including high-resolution functional brain imaging and devices which purport to transduce information from a lost sensory modality into the brain via another modality, inroads are being made toward finding answers to these questions. Sensory substitution devices (SSDs) aim to compensate for the loss of a sensory modality, typically vision, by converting information from the lost modality into stimuli in a remaining modality (Bach-y-Rita and Kercel, 2003). Here we utilized sensory substitution to examine how the very first stages of learning to “see with sound” occurs, and the quality of the information transfer from vision to audition as assessed with a test of acuity. A more complete understanding of the way in which this occurs may assist in the development of such devices that not only replicate lost sensory functionality, particularly in the blind, but along with research on synesthesia and multisensory processing, also call into question our notion of sensory modalities as functionally discrete, non-overlapping entities.

### Changes following sensory loss

Major neuroplastic changes can occur in a brain that is undamaged but loses input from a sensory modality. Multisensory processes in which cues from multiple modalities unite to form a percept may also include a degree of redundancy: an object's shape can be discerned by the hands and eyes simultaneously or separately; the eyes and ears can be used in concert to determine the direction of a physical sound source more accurately than from sound alone. It may be this redundancy which helps the brain to compensate for sensory loss by enhancement of function of the remaining senses (Merabet and Pascual-Leone, 2010).

Blind individuals, particularly those born without sight or those who lost sight early in life often show superior performance in other modalities, including finer pitch discrimination and sound localization, more accurate tactile discrimination, better speech discrimination, and verbal recall (Merabet et al., 2005; Pasqualotto and Proulx, 2012; Pasqualotto et al., 2013). Blind individuals lack normal visual input to their occipital cortices, but brain imaging studies have shown that this area is nevertheless active during a number of tasks, including Braille reading, auditory localization tasks, speech comprehension, and verbal recall (Merabet et al., 2005).

Sensory loss need not have occurred early in life however, and changes can occur rapidly in adults following sensory deprivation. In one study, participants were blindfolded 24 h per day for 5 days and given intensive training in tactile and spatial discrimination tasks. Participants experienced visual hallucinations soon after blindfolding and functional magnetic resonance imaging (fMRI) scans showed occipital cortex activation when fingertips were stimulated, with primary and secondary visual cortices becoming increasingly active over the blindfolded period (Pascual-Leone and Hamilton, 2001). Tactile discrimination skills learnt during the experiment were disrupted when participants' occipital cortices were subjected to repetitive transcranial magnetic stimulation (rTMS). After the blindfold period, participants' brains were scanned again and occipital cortex activity linked to tactile stimulation was absent. Because the changes seen in this experiment manifested and then reversed so quickly, it cannot have been that new neuronal connections were established. Instead, existing connections between somatosensory, auditory, and visual cortices were “unmasked” when input from the eyes temporarily ceased. The authors of this study suggest that neuroplasticity in response to sensory loss is a two-stage process: rapid unmasking of existing cortico-cortical connections followed by slower and more permanent formation of new neuronal connections (Pascual-Leone and Hamilton, 2001).

### Sensory substitution

Sensory substitution is the use of one modality (the substituting modality) to take the place of another (the substituted modality). The concept has been construed by some in a very broad sense to include, for example, reading, in which vision (the written word) takes the place of audition (the spoken word) (Bach-y-Rita and Kercel, 2003). More commonly, however, the term is used to refer to a means to allow a person who has suffered sensory loss to make use of their remaining senses to perform functions normally carried out using the lost sense. An obvious and widely used example of this is Braille, in which tactile perception via the fingers substitutes for vision (or, arguably, audition), allowing blind people to read. This system only replaces a specific aspect of a modality however, namely language; substitution on a general level represents a much greater technical challenge. This challenge has been met over the past four decades by a variety of systems and devices, most of which have been designed to replace vision, either by touch or audition.

### Auditory-visual sensory substitution

In tactile-visual sensory substitution (TVSS) systems, the skin or tongue functions as an analog of the retina (Bach-y-Rita et al., 1969). However, by comparison it is very crude and low-resolution. Kokjer (1987) estimated the informational capacity of the human fingertip to be in the order of 100 bps. The eye, by comparison, has been estimated to deliver around 4.3 × 10^6^ bps (Jacobson, 1951), some four orders of magnitude greater bandwidth. The ear falls between these two limits, its capacity has been estimated at around 10^4^ bps (Jacobson, 1950). So although parallels between the auditory and visual systems are not obvious in the way that the skin/retina analog is, the ear has the potential to provide a higher-throughput means of directing visual information to the brain than the skin.

The first general-purpose auditory-visual sensory substitution (AVSS) system was developed by Meijer (1992). It is known as “The vOICe” and is the system used in the present study. The vOICe converts images captured by a camera into “soundscapes” delivered to the user through headphones at a default rate of one soundscape per second. Each soundscape is a left to right scan of the visual scene with frequency representing the image's vertical axis and loudness representing brightness (these mappings are not arbitrary, see Evans and Treisman, 2010). The user therefore experiences a series of “snapshots” passing from the left to the right ear. Other AVSS devices have been developed: one which uses a similar encoding protocol as The vOICe but converts scenes into images resembling line drawings and produces a more “musical” output (Cronly-Dillon et al., 1999, 2000); another, the Prosthesis for Substitution of Vision by Audition (PVSA), does not scan the visual field but lets frequency increase both from bottom to top and from left to right of the captured image, using a higher density of auditory “pixels” in the center of the image to simulate the fovea (Capelle et al., 1998); and a third, the Vibe, also does not scan the visual field, instead dividing it into several “receptive fields” which are presented simultaneously, their position encoded by frequency and left-right audio channel balance (Auvray et al., 2005; Hanneton et al., 2010).

As with TVSS devices, users of AVSS systems report distal attribution (Auvray et al., 2005). Users have been shown to recognize patterns (Arno et al., 2001), recognize and locate objects in 3D space, including placing previously unseen objects into categories, such as “plant” or “boot” (Auvray et al., 2007; Merabet et al., 2009). One expert late-blind user of The vOICe, P.F., has provided repeated, detailed descriptions of her experiences which, she claims, have gradually improved and become more like vision. Depth perception, smooth movement (as opposed to 1 Hz “snapshots”) and even experience of colors emerged with continued use of the device for P.F., suggesting that her brain had been gradually adapting to more efficiently process this novel kind of auditory information (Ward and Meijer, 2010).

### Acuity in sensory substitution systems

An important factor in the usefulness of a system in which vision is the substituted modality is the limit on detail resolvable by the user. Finding this limit can be achieved in much the same way that visual acuity is conventionally measured. Some studies have measured acuity through indirect means, by assessing the ability of participants to either localize or recognize objects with AVSS devices (Auvray et al., 2007; Proulx et al., 2008; Brown et al., 2011). The study by Proulx et al. (2008) even used an ophthalmic perimeter, commonly used to map the visual field, as a means of assessing the speed and accuracy of spatial localization using sensory substitution. Other studies determined the acuity limits of TVSS devices directly. The acuity limit of legal blindness in the United States is 20/200; that is, a person with this level of acuity can read an eye chart located 20 feet away as well as a person with normal vision would read the same eye chart were it 200 feet away (Social Security Act. United States Social Security Administration, 2006). Normal vision thus corresponds to an acuity of 20/20.

The translation of visual acuity to sensory substitution is not entirely straightforward as the computation requires consideration of the field of view provided by the device. For example, it might be physically possible to provide 20/20 vision with a SSD through telescopic means. However, if this is accompanied by severe tunnel vision due to a restricted field of view, then the end result is still classified as a severe visual impairment. In fact, the definition of legal blindness in the United States specifies an acuity of 20/200 or less, or a field of view of 20° or less. A full explanation and demonstrations of the issues involved in defining acuity for sensory substitution are also available online ^1^, but we will summarize the main points here. For our calculations of acuity we will assume a 60° field of view for the camera, as we used in the experiments reported here. This is a typical field of view for web-cams and similar devices, (and may, for lack of a suitable standard, serve as a ballpark figure for a practical field of view). The minimum number of pixels required to portray most optotypes used in acuity measurement would be 2–3 pixels horizontally. Assuming 176 horizontal pixels for the camera input, as we also use in our experiments, then every pixel subtends approximately 0.35° in width. The smallest discernable optotype then spans about one degree for 3 pixels horizontally (3 × 60°/176≅1°), or 0.7° for 2 pixels (2 × 60°/176). Normal vision under Snellen's definition is the ability to recognize one of the Snellen chart optotypes when it subtends 5 min of arc (Duke-Elder and Abrams, 1970). Functionally, this means that visual acuity under the above conditions is between 8 and 12 times less than that possible with normal human vision. At best, visual acuity could be in the range 20/160–20/240. The crucial aspect of these calculations for comparisons with reports of visual acuity in the literature is that they are based on a horizontal resolution of 176 pixels for a 60° field of view. If the physical resolution of a sensory device provides much less than this, then the maximum visual acuity possible with that device is dramatically reduced for the same field of view.

The first study to assess visual acuity with sensory substitution was conducted in the domain of touch by Sampaio et al. (2001). Sampaio et al., used the Snellen tumbling E paradigm to test blind and sighted naïve participants' performance using a 3 cm^2^ 12 × 12 electrotactile array or “tongue-display unit (TDU).” Their setup included a camera with a 54° horizontal and 40° vertical field of view, and its 280 × 180 pixel frames were down-sampled to the 12 × 12 tactile display resolution by averaging adjacent pixels. Judging acuity as performance at or near 100% in letter orientation discrimination they reported that all participants were able to achieve this to an acuity level of 20/860 and that two participants of median performance doubled their acuity after 9 h of training to 20/430. Because the device provided a resolution of 12 pixels horizontally, the actual functional acuity might be far less, with a maximum theoretical acuity of 20/2400 for a 2 pixel wide optotype and a 60° field of view, or 20/2160 when calculated for their camera's 54° field of view. For example, in the latter case the denominator is calculated as (2 pixels × 54°/12 electrodes) × (60 min of arc per degree/5 min of arc for normal vision) × 20 for normal vision = 2160.

The second study to assess acuity was conducted by Chebat et al. (2007), who tested a larger sample of early blind and sighted participants on a 4 cm^2^ 10 × 10-array TDU. After a period of training participants were tested also using the Snellen tumbling E. The criterion for passing each level was 70% correct responses. Acuity scores ranged between 20/1800 and 20/8400 for an estimated 29° field of view, and it was found that blind participants were overrepresented at higher acuity scores with 8.4% of sighted and 31.3% of blind participants achieving the highest score. Again, by using the calculations and limitations described earlier, the maximum theoretical acuity for a 10 pixel device such as this would be 20/2880 for a 2 pixel wide optotype and a 60° field of view, or 20/1392 when calculated for their 29° field of view. The latter is consistent with the range of acuity scores reported by Chebat et al. (2007) for their narrower field of view.

Acuity using The vOICe AVSS device has recently been reported by Striem-Amit et al. (2012) for nine fully blind participants who had already been trained to use the device. Participants were trained for between 55 and 101 h and tested on Snellen tumbling Es. Using a criterion of 60% correct responses, participants' acuity is reported to have varied between 20/200 and 20/600 using a 66° field of view camera. The present study was designed to assess a number of additional issues beyond the scope of the study by Striem-Amit et al. First, their study was conducted only with expert users of the SSD who were also blind. It is thus unclear whether the acuity levels achieved reveal the resolution of the device, or rather the compensatory neural plasticity of the blind participants combined with their expertise in using the device. Furthermore, the mechanisms that give rise to the acuity performance are also unclear. To provide a benchmark measure of acuity, we here employed naïve sighted participants without previous experience of the device. Furthermore we tested them under different conditions (static and active use of a camera), and with additional experiments and questionnaires to determine the possible correlates of individual differences in acuity performance.

The present study also used the Snellen tumbling E in two separate experiments: in the first, The vOICe software was used to turn letter images of decreasing size into sound files offline which were played to participants as static soundscapes; in the second, blindfolded participants used a sunglasses-mounted camera and headphones to “read” letters from a screen. Acuity in present tongue-based TVSS devices is limited by the number of electrodes on the array (144 in Sampaio et al., 2001 and 100 in Chebat et al., 2007). The vOICe software, by contrast, produces an equivalent resolution of 11,264 “voicels” or auditory pixels in the default setting. This fact, along with the higher informational capacity of the ear (Jacobson, 1951) suggests that higher acuity scores with audition should be possible than those in the tactile studies cited above (see, e.g., Sampaio et al., 2001; Chebat et al., 2007; Striem-Amit et al., 2012).

As well as assessing the mean acuity of a sample group, the present study also takes an individual differences approach to determine whether any correlations can be found between performance on acuity tests with a SSD and other metrics. It has been shown, for example, that musical training correlates with improved ability to extract information from sound pre-attentively (Koelsch et al., 1999), and to extract speech from noise (Parbery-Clark et al., 2009). Many of the participants also took part in additional experiments to explore such individual differences. First we assessed whether there was any relationship between acuity and another form of auditory expertise, musical training. Their ability to discriminate between similar musical phrases and their pitch discrimination abilities was also tested. This study also considered whether early, naïve use of sensory substitution immediately draws upon the substituted modality (vision) or only the substituting modality (hearing). Work on synesthesia, a cross-wiring of the senses where a sound might evoke a visual experience, such as music evoking different colors (Hubbard et al., 2011), suggests that the sensory modalities are not always distinct, independent modules. Certainly one broad goal for work on sensory substitution is to ultimately provide the phenomenological experience of vision in a form of synthetic synesthesia (Proulx and Stoerig, 2006; Proulx, 2010). Along this line of interest, here participants also took a vividness of visual imagery questionnaire (Marks, 1973), as well as a psychophysical test designed by Cui et al. (2007) to correlate with the vividness of imagery reported by participants. If individual differences such as these can be found to correlate with acuity performance they may be useful as proxies to gage a person's likelihood of making successful use of an AVSS device such as The vOICe and to reveal potential mechanisms for such performance. This also assesses whether visual imagery evoked by the device, as a form or synthetic synesthesia (Proulx and Stoerig, 2006; Proulx, 2010), is related to measures of the functional resolution possible with the device.

## Experiment 1

### Participants

Adult volunteers without experience with The vOICe took part (4 male, 22 female, mean age 22.6 years, range 19–32 years). All reported normal vision (with corrective lenses in some cases).

### Apparatus

For Experiment 1a Dell Optiplex 760 PC (Intel Core 2 Duo @3 GHz; 3.2 GB RAM; Microsoft Windows XP Professional) ran The vOICe software (Learning Edition v1.91) ^2^, with the foveal enlargement mapping disabled. The program was run on “slow motion” setting, images being scanned from left to right producing soundscapes with a duration of 2 s, and in “negative video” mode whereby dark areas correspond to loud sounds and white areas produce no sound. Sennheiser HD555 open-back supra-aural headphones were used for all tests involving an auditory component. The program's foveal enlargement option was kept disabled in all experiments.

For Experiment 1b FrACT visual acuity software (Bach, 1996; v3.7.1b, obtained from michaelbach.de/fract/download.html) was used, running the tumbling E experiment. Four orientations and differing sizes of the letter E in black on a white background were shown on an LCD screen with resolution 1440 × 900, and each image was followed by a 200 ms mask. Participants sat 175 cm from the monitor. All instructions and requirements were followed according to the FrACT specifications.

### Materials

**Figure 1:**
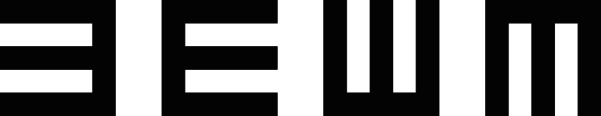
**Four orientations of Snellen E converted to “soundscape” stimuli: left, right, up, down**.

Digital images of the Snellen E in four orientations (left, right, up, and down, Figure 1) and 10 sizes (Table 1) were converted by The vOICe software first into a 176 × 64 pixel resolution and then into soundscapes, with the optotypes set as white to be sonified and the background black and silent. These values were used to calculate the Snellen acuity for each letter size following procedure detailed on The vOICe website (see text footnote 1), assuming a 66° camera field of view as used in Experiment 2. Optotypes have also been assigned an estimated “width” in mm assuming a 66° camera viewing angle at a distance of 1 m in order to compare results with those of Striem-Amit et al. (2012) (Table 1). A questionnaire about the experience of using The vOICe software and any strategies employed by participants to detect optotype direction was used.

**Table 1:** **Size and acuity values of the optotypes used in each block**.

**Block**	**Snellen acuity**	**Original letter size (pixels)**	**Width of letters after vOICe conversion (pixels)**	**“Width” of letters with 66 camera angle at 1 m (mm)**
1	20/13965	500	170	1255
2	20/11583	416	141	1041
3	20/9365	336	114	841
4	20/6982	250	85	627
5	20/4682	165	57	421
6	20/2464	85	30	221
7	20/1882	65	23	170
8	20/1308	45	16	118
9	20/737	25	9	66
10	20/408	15	5	37

### Procedure

The concept of The vOICe was explained to participants and they were asked to read an explanation of the image-to-sound conversion protocol and the experimental procedure. They were asked if they understood what they had read and that they consented to taking part in the experiment. They were then asked put on a blindfold and headphones. The first experiment took a total of 40 min per participant.

#### Experiment 1a

The experiment was conducted as blocks of trials with 12 trials per block. In each trial the soundscape was played to the participant, who had to state which direction they thought the optotype was facing (i.e., the tines of the E, see Figure 1). They were allowed to ask for the soundscape to be repeated up to ten times. Opto-types were presented in pseudorandom order with each direction featuring three times. The threshold for passing each block was 9/12 correct optotype directions, with the exception of Block 1 which they could repeat up to five times if they failed to reach the threshold (with different orders of optotypes each time). Thereafter blocks were presented in order until the threshold was not reached at which point the experiment ended and the highest successfully completed block was recorded as the participant's vOICe acuity score.

#### Experiment 1b

Participants then took the FrACT visual acuity test. The test was explained to them and they were asked to sit 1.75 m away from the computer screen. They called out the direction of each optotype presented which was entered into the computer by the experimenter who sat behind the display and thus did not have exposure to the stimuli. When the test was complete the Snellen fraction displayed on the screen was recorded as their visual acuity score. The test consisted of 30 trials. Finally, participants were asked to fill in a questionnaire about their experience.

### Experiment 1A RESULTS

**Figure 2:**
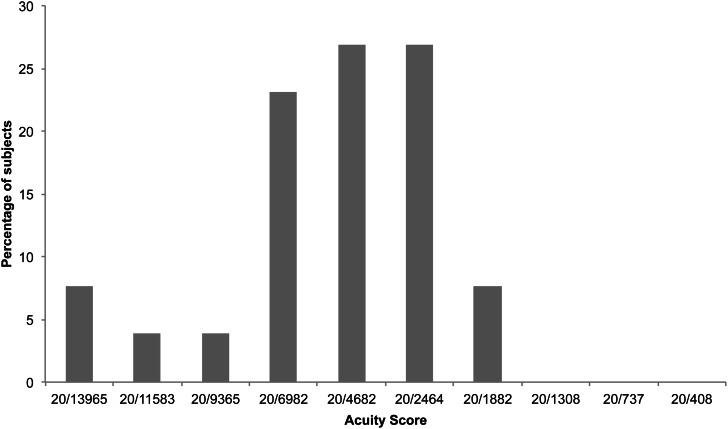
**Percentage of participants who achieved each vOICe acuity score (9/12 correct responses) in experiment 1**.

Participants (*n* = 26) achieved vOICe acuity scores between 20/13965 and 20/1882. Scores of 20/2464 and 20/4682 were achieved by the highest number of participants (Figure 2). Median acuity was 20/4682. Thirteen participants completed the first block on the first attempt, nine on the second attempt, three on the third attempt, and one on the fourth attempt. There was a non-significant negative correlation between number of Block 1 attempts and final acuity score (Spearman's rank correlation *r*_*s*_ = −0.37, *p* = 0.07).

### Experiment 1B results

Median visual acuity as measured by the FrACT test was 20/13. No correlation was found between visual acuity and vOICe acuity. Participants who had musical experience achieved higher vOICe acuity scores (Mann–Whitney test, *U* = 19.5, *p* = 0.03, *r* = 0.43).

### Experiment 1 questionnaire results

Participants were first asked about the experience of using The vOICe for the acuity task: “How would you describe your experience of the task? Can you compare it to a sensory modality/experience.” A fifth of the participants described the auditory nature of the tasks (19%); for example, one participant noted its similarity to a music test. A few also noted that it felt like they used a general spatial sense to carry out the task (15%), interestingly described by one as “Like figuring out where you're walking in the dark.” Nearly one-third mentioned experiences in the visual modality (30%), such as visualizing or imagining the letter E while trying to complete the task.

Participants were next asked about any strategies used to complete the task: “Did you use a particular strategy to identify the different orientations? Can you describe this strategy?” Half provided very specific, detailed strategies that were used, while half provided just a vague description. All responses were classifiable as having relation to either determining an orientation difference, using visual descriptions, and whether the participants attempted to either memorize the sounds, work out how the sounds related to the image that produced them, or actively tried to imagine the stimuli visually. First, 58% reported a strategy of determining the orientation difference. This is noteworthy considering the task could have been approached as an auditory discrimination task that did not have anything orientation-specific about it. Only 23% reported using an explicitly “visual” strategy, however, and instead spoke of the sounds as having a spatial quality that was implicitly related to the visual image; for example, one participant noted that the sounds had sides, rather than beginnings and ends (emphasis added) “The pitch change at either *side* of the sound indicated a left or right orientation.” The task was not automatically carried out, as 61% of the participants noted they had to deliberately work out the response after listening to the sounds. Another 15% said that they attempted to memorize the sounds and the correct response; a difficult feat considering that the range and modulation of pitch changed as a function of image size, thus making the sounds different at each level of acuity. However 23% attempted to imagine the image of the E that created the sound in order to respond.

## Experiment 2

### Participants

A subset of volunteers from Experiment 1 returned for additional experiments (2 male, 15 female, mean age 23 years, range 20–32 years). Procedures were approved by the Queen Mary Research Ethics Committee, and all participants gave informed, written consent.

### Apparatus

“Spy” sunglasses (Mini DVR Spy sunglasses, Camera Audio Video Recorder, Multister, Hong Kong) with an image sensor similar to a webcam built into the bridge were used, in conjunction with The vOICe software, to convert Snellen Es displayed on a 1440 × 900 LCD screen into soundscapes as participants faced the screen. Movement was possible, thus the participants may have centered the stimuli however they preferred.

### Materials

#### Experiment 2

The same optotype soundscapes were used as in Experiment 1. For the part of the experiment in which subjects wore camera glasses, optotypes were displayed on screen in white on black. The same sizes (original, in pixels) as those shown in Table 1 were used. Participants sat so that the camera glasses were 30.5 cm from the screen. At this distance a Block 1 letter occupied the equivalent area of the camera's field of view as a Block 2 letter occupied of The vOICe's “screen” size (i.e., 141 pixels out of 176 on the horizontal axis). Accordingly, in this part of the experiment the first block represented the same acuity score as Block 2 in Table 1, therefore there were nine blocks in this part of the experiment.

### Procedure

#### Experiment 2

The same procedure as Experiment 1 was carried out for the optotype soundscapes, except in this case participants completed all blocks regardless of their performance. Experiments 2, 3, and 4 were all carried out in a single session, approximately 2 months after Experiment 1 was conducted, and took approximately 60–70 min per participant.

### Experiment 2 results

**Figure 3:**
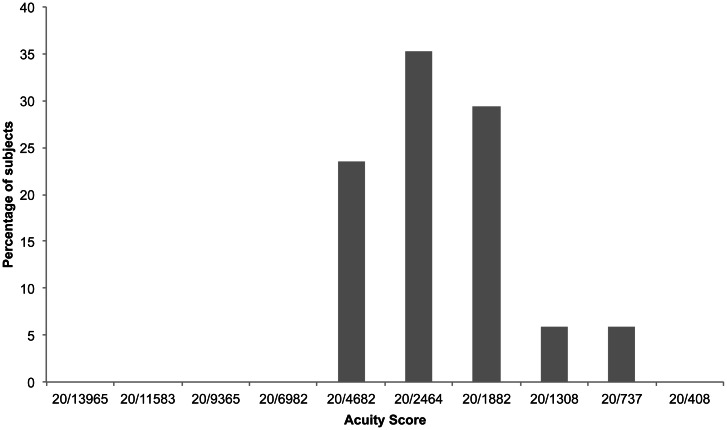
**Percentage of participants who achieved each vOICe acuity score (9/12 correct responses) in experiment 2**.

**Figure 4:**
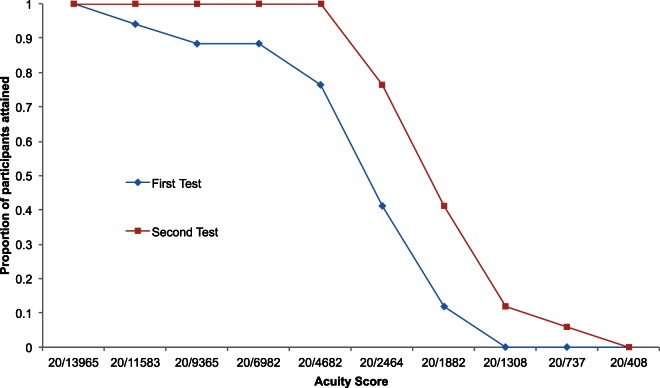
**Difference in participants' performance between experiment 1 and experiment 2.** Proportion of participants achieving each acuity score (9/12 correct responses) is shown as a function of increasing acuity score.

Participants (*n* = 17) achieved vOICe acuity scores between 20/4682 and 20/737 (based on the first experimental criterion of 9/12 correct responses). A score of 20/2464 was achieved by the highest number of participants (Figure 3). Median acuity was 20/2464. Participants who took part in both experiments achieved higher acuity scores in the second test (Wilcoxon signed ranks test, *Z* = −2.99, *p* = 0.002; Figure 4), with the group as a whole improving from an acuity level of 5 on the first test to over level 6 on the second (6.3). Two individuals who had only reached levels 1 and 2 during the first experiment were able to attain levels 5 and 7 in the second.

**Figure 5:**
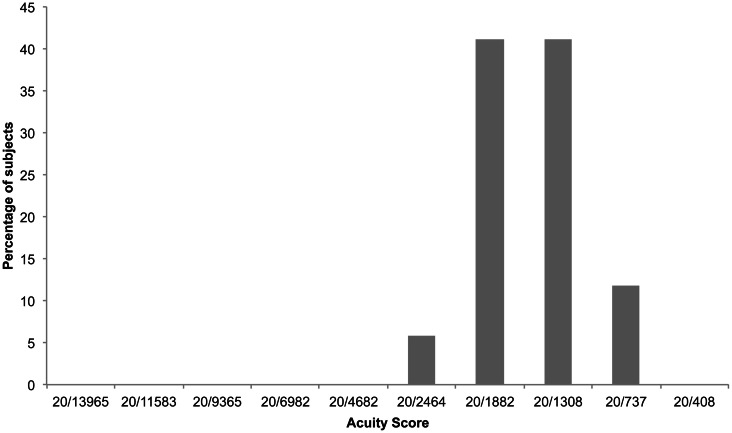
**Percentage of participants who achieved each vOICe acuity score (8/12 correct responses) in experiment 2**.

**Figure 6:**
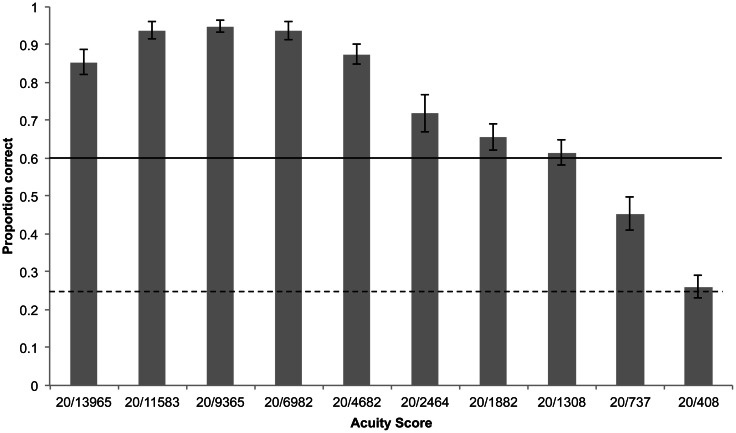
**Mean proportion of correctly identified static optotype orientations by all participants for each block, shown on the X-axis as its corresponding acuity score.** Error bars represent standard error of the mean, horizontal solid line represents threshold of 0.6 (8/12 correct responses), dashed line represents chance performance.

Using the same experimental criterion as Striem-Amit et al. (2012) of 8/12 correct responses, participants achieved scores between 20/2464 and 20/737. Scores of 20/1882 and 20/1308 were achieved by the highest number of participants (Figure 5). Median acuity was 20/1308 Participants collectively were significantly above the threshold of 8/12 correct responses up to 20/2464 and above chance (4/12) up to 20/737 (Figure 6).

**Figure 7:**
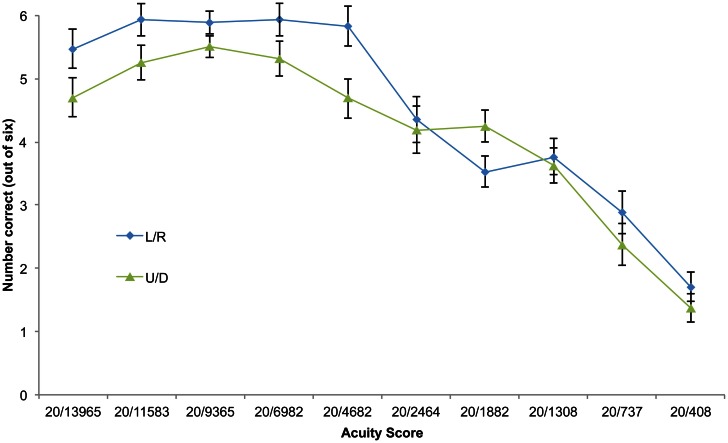
**Difference in performance between up/down (U/D) and left/right (L/R) static optotype orientation.** Orientation categories shown as functions of increasing acuity score (correct responses out of six, i.e., half the total number of trials per block). Error bars represent standard error of the mean.

Performing a two-way analysis of variance (ANOVA) on ranked acuity scores showed significant main effects of size (*F*(1,9) = 14.3, *p* = 0.004) and of orientation (*F*(9,9) = 6.25, *p* = 0.03). There appeared to be an interaction between size and orientation such that at large sizes performance was superior on left/right oriented optotypes, and at acuity level 20/1882 performance was superior on up/down oriented opto-types (Figure 7), however the interaction effect was not significant.

**Figure 8:**
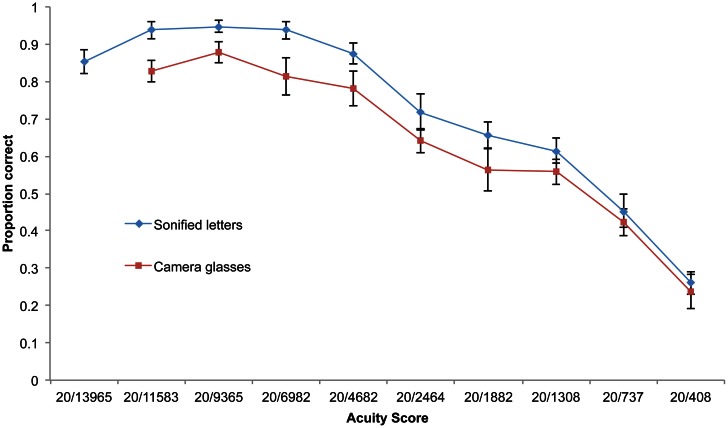
**Performance on each acuity category shown as a function of increasing acuity score for static letters and use of camera glasses to sonify letters on screen in real time.** Error bars represent standard error of the mean.

**Figure 9:**
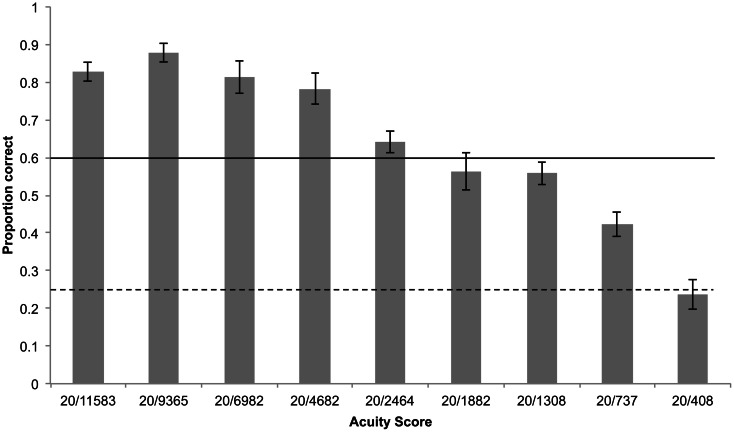
**Mean proportion of correctly identified optotype orientations using camera glasses by all participants for each block, shown on the X-axis as its corresponding acuity score.** Error bars represent standard error of the mean, horizontal solid line represents threshold of 0.6 (8/12 correct responses), dashed line represents chance performance.

Participants' (*n* = 15) acuity when tested on the static soundscapes was slightly but not significantly higher than when using camera glasses to view optotypes (Wilcoxon signed ranks test, *Z* = −1.56, *p* = 0.06; Figure 8). Participants were significantly above the 8/12 threshold up to 20/4862 and above chance (4/12) up to 20/737 (Figure 9).

## Experiment 3

### Participants

The same subset of volunteers in Experiment 2 from Experiment 1 returned for this experiment (2 male, 15 female, mean age 23 years, range 20–32 years). Procedures were approved by the Queen Mary Research Ethics Committee, and all participants gave informed, written consent.

### Apparatus

#### Experiment 3

MATLAB R2009a (v7.8.0) software with Psychophysics Toolbox extension (Brainard, 1997; Pelli, 1997) was used to run a Stroop-style psychophysical test “EagleStroop” (Cui et al., 2007; used with permission) in which a 32 ms presentation of a colored background (orange, yellow, purple) was followed by a 32 ms appearance of one of three corresponding color words (in black) or no word. Color words were sometimes congruent with preceding background color. The test was presented on an 1440 × 900 LCD screen, as also used in Experiment 2.

### Procedure

#### Experiment 3

The “EagleStroop” psychophysical test was carried out, consisting of five practice trials in which colored backgrounds and color words appeared for 1000 ms each, followed by 120 recorded trials in which stimuli appeared for 32 ms. Participants entered the initial letter of the color word they thought they had seen by pressing that key on the computer keyboard or pressed the space bar if they saw no word. Scores were automatically recorded by the MATLAB software and the difference between correct responses when color and word were congruent and when incongruent for each participant were calculated.

### Experiment 3 results

No significant correlations were found between vividness of imagery as measured either by the vividness of visual imagery questionnaire (VVIQ) test or the “EagleStroop” psychophysical test and vOICe acuity; nor was a correlation found between VVIQ scores and the EagleStroop test. On the EagleStroop test there was no difference in performance when color and word were congruent and when they were incongruent across participants although there were differences in individual participants.

## Experiment 4

### Participants

The same subset of volunteers in Experiments 2 and 3, originally from Experiment 1, returned for this experiment (2 male, 15 female, mean age 23 years, range 20–32 years). Procedures were approved by the Queen Mary Research Ethics Committee, and all participants gave informed, written consent.

### Apparatus

#### Experiment 4

Participants' ability to discern pitch and to differentiate between similar musical phrases was carried out using online tests on the Tonometric website^3^. The “adaptive pitch” and “tonedeaf” tests were used.

### Materials

A “Vividness of Visual Imagery Questionnaire” (Marks, 1973) was used.

### Procedure

#### Experiment 4

**Figure 10:**
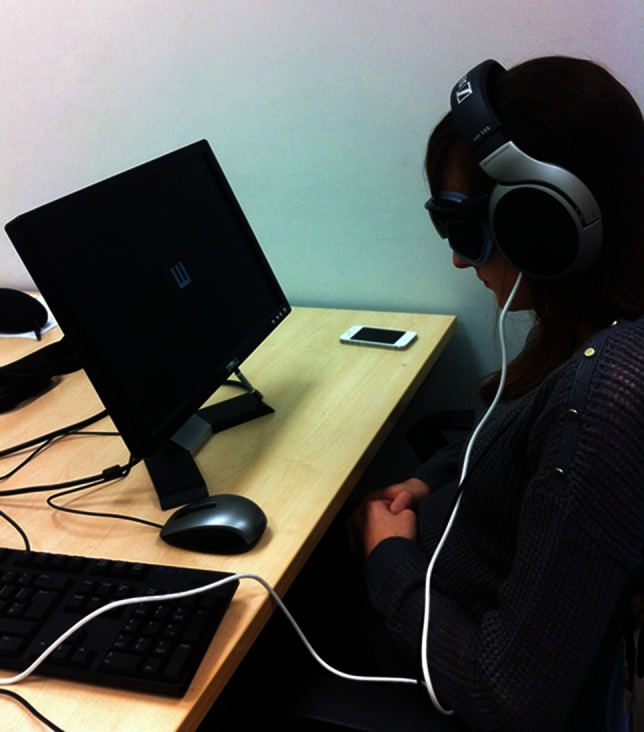
**Participant wearing camera glasses and listening to the soundscape produced from the camera's view of the optotype displayed on screen**.

For the “Tonometric” auditory perception tests, participants wore headphones and used the computer mouse to enter “higher” or “lower” in each trial of the “adaptive pitch” test, and “same” or “different” for the “tonedeaf” test. Their scores were recorded. For the next part of the experiment, the use of camera glasses in conjunction with The vOICe software was explained to participants. They were then asked to put on a blindfold, followed by the camera glasses which were connected to the computer by a USB cable. Headphones were placed on top to keep the glasses fixed in place. They were seated so the image sensor was 30.5 cm from the computer screen and asked to keep their head as still as possible but centering the stimuli as they preferred (Figure 10). Optotypes were then displayed on the screen and the same protocol was used as with the previous part of the experiment in which soundscapes were played, except that as The vOICe software was converting images to sound “live,” the soundscapes played repeatedly until the participant stated the direction of the optotype. Finally, participants were then given a copy of the VVIQ and instructions on how to complete it.

### Experiment 4 results

On the Tonometric tests of auditory perception participants' mean pitch discernment was 8.17 Hz at 500 Hz (SD = 9.00, *n* = 16). Their mean score on the “tonedeaf” test was 72.73% correct (SD = 8.86, *n* = 17). No significant correlations were found between vOICe acuity score (static letter soundscapes) and either of these tests.

## Discussion

The results of this study demonstrate firstly that very little training or explanation is required to carry out tests of this nature: every participant understood the concept of The vOICe, even if it was an entirely novel one to them. Half of the participants in Experiment 1 (13 of 26) completed the first block without needing to repeat it and no participant failed this initial training session. There was no link between performance on The vOICe acuity test and participants' actual visual acuity.

The repeat of the original vOICe test (Experiment 2), although it took place around 2 months after the first and without further training (Experiment 1), revealed a significant improvement in performance amongst participants who returned; there is no reason to suspect any link between initial performance and likelihood of returning. Returning participants needed little reminding of the procedure, and only one of the 17 required more than one attempt at the first block. Furthermore, all participants achieved a score of 20/4682 or greater (Figure 3), in contrast to the first experiment in which all scores below 20/4682 were represented by at least one participant (using the same criterion of 9 correct trials out of 12; Figure 2). The final acuity category of 20/408 appears to represent the upper limit of performance for all participants with scores in both iterations of this test no better than chance (Figure 4). Given that the optotypes were reduced to 5 pixels in width this limitation is likely explained by the physical representation provided being a sub-sampling of the original image. This makes it even more impressive that some participants were able to discriminate the orientation of the stimulus with only two vertical pixels being sonified. It is interesting to compare this to the study by Sampaio et al. (2001), where one blind and one sighted participant each received 9 h of training in using the TDU; they both doubled the acuity level after that training. Here, without additional training and a 2 month gap, we found that two participants went from basic level (1 and 2) performance in the first attempt, improved to higher levels (5 and 7, respectively) in the second attempt.

These results are comparable with tongue-based tactile acuity tests carried out on 15 blind and 25 sighted participants by Chebat et al. (2007). Using a criterion of 70% correct responses per acuity category, they reported acuity scores ranging between 20/1800 and 20/8400 for a 29° field of view, with most sighted participants scoring 20/3600. As noted in the Introduction, if these reported scores are corrected for the limited field of view provided, then the maximum acuity of 20/1800 would be calculated as approximately 20/3600 in a manner most comparable to the scores with The vOICe reported here where we found a maximum of 20/408 for a 60° field of view. The present study's slightly more stringent criterion of 75% correct saw most participants scoring 20/2464 or 20/4682 in Experiment 1. An earlier study (Sampaio et al., 2001) found considerably higher acuity among blind and sighted participants than the later study by Chebat et al. (2007), but from the estimates for maximum theoretical acuity we suspect errors in those early results. Acuity scores in the present study were not as high as those reported by Striem-Amit et al. (2012) although there is some overlap: the lowest score found in their study was 20/1000 and the highest was 20/200, which equates to optotypes of width 73 mm and 15 mm respectively at a distance of 1 m. One possible explanation is that the limitation with the smallest optotype presented at 5 pixels by 2 pixels may not have been present in that study. Moreover, participants in that study had received a great deal of training (over 100 h in some cases), and thus perceptual learning processes might further account for the performance difference (Proulx et al., 2013). Although Stiem-Amit et al. do not report any correlation between training time and final performance, the difference in performance between the first and second acuity test in the present study shows a clear effect of training, which in this case amounted to <1% of that received by participants in the study by Striem-Amit et al. (2012). Also, all of their participants were blind, all but one of them congenitally, and this may also have been a factor in their high performance (see Chebat et al., 2007).

Interestingly, the orientation of the optotypes affected performance and did so differentially depending on the size of the optotype. When optotypes were large participants found it easier to discriminate left and right; as they became smaller, up/down discriminations briefly became easier even with the greater reduction in the number of pixels representing the vertical dimension (see Figure 7). The difference between a left and right-facing E is in the temporal dimension; when the E is facing up or down, differences are distinguishable by pitch. Because this pitch difference continues for the time corresponding to the entire width of the E, it is presumably easier to detect when optotypes are smaller, whereas the difference between left and right exists for only one fifth of this time. This is a property of The vOICe and is in no way comparable with TVSS devices. It would be very interesting for future studies to go further in determining whether differences exist between these temporal and pitch-based components of auditory acuity, both between and within individuals. If significant differences are found within individuals, this may be used as a basis for tailoring The vOICe to an individual user's own capabilities in these two separate abilities. Furthermore, it would be interesting to see whether this differential ease-of-discernment bias continues to hold as users become more proficient with training. Alternatively, it may be possible to eliminate this bias by using other optotypes; for example, Snellen Es angled at 45° or Landolt Cs (see Bach, 1996). This would also provide a test of how well the acuity of the device generalizes to other measures and features.

There was no statistically significant difference between acuity scores using static soundscapes and using camera glasses to “view” the letters (see Figures 9 and 10). Performance using the camera glasses may be expected to be slightly impaired, firstly because this was each participant's first attempt at using this equipment and secondly because “live” sonifications will inevitably be “rougher” than those created as static soundscapes, due to inconsistencies in lighting, movements made by participants (although they were instructed to remain as still as possible). However the use of a live camera feed should also provide superior resolution by allowing a dynamic sub-pixel displacement with the camera view, as well as a representation of the stimulus via different frequency ranges. The anti-aliasing of the image due to the representation in pixels would change with movement and perhaps provide a clearer view of the optotype. Also it might be easier to discriminate spectral differences in the higher frequency range than in the lower range, and moving the camera would allow for such sampling by elevation.

A link was found in Experiment 1b between participants' reported musical experience and their vOICe acuity: those with experience attained higher scores. An attempt was made to further subdivide participants into those with some experience and those with extensive experience but significance was lost, which may have been due to the small sizes of these resultant categories. However, there were no significant correlations between vOICe acuity and either pitch discrimination or the ability to discriminate between two similar musical phrases. It might tentatively be inferred therefore, that it is musical training, or experience in fine sound discrimination, that makes a difference in this test of auditory acuity, rather than aptitude. In fact most reported some experience, although without more detailed questioning it is difficult to quantify the degree of their training, so results should be treated with caution. They do accord with previous studies linking musical experience with improved auditory skills (Koelsch et al., 1999; Parbery-Clark et al., 2009).

There was no link between participants' subjective assessment of the vividness of their visual imagery and their vOICe acuity scores. A major drawback with using a questionnaire to measure this is inter-subject comparability, due to the highly subjective nature of the measure. A given individual may believe her visual imagination to be very vivid, perhaps because she can remember specific details of a familiar scene, but has no reliable way of comparing it with that of another who may actually imagine that scene in much more visual-phenomenological way. Cui et al. (2007) reported a correlation between subjective vividness of imagery and performance on their Stroop-style psychophysical test using a sample size of 8 and concluded that it could be used as an objective measure of vividness of imagery. The present study failed to replicate this result using a sample size of 15, calling that conclusion into question.

The approach of assessing the perceptual resolution, or acuity, of sensory substitution suggests that such assessment might be made in other areas of research. For example, the resolution at which attention operates has become a recent area of interest (He et al., 1996, 1997; Intriligator and Cavanagh, 2001; Moore et al., 2008). Such an approach can determine the precision with which sensory information can be processed by the different modalities, and for different functions. Measures of resolution such as acuity might also provide a benchmark measure for comparing sensory substitution algorithms, sensory modalities, and also might predict individual differences in sensory substitution acquisition. Considering that some long-term users of sensory substitution experience a form of synthetic synesthesia (Ward and Meijer, 2010), it might also be possible to assess the resolution and precision of synesthetic experiences. For example, when a sound evokes the perception of color for a synesthete, what is the spatial extent of that experience? How constrained is the experience by color, size, and shape? The investigation of the resolution of perceptual experience can provide important information about the nature of information processing across and within sensory modalities, and the further exploration of this issue for sensory substitution and other areas of perception will help to reveal the underlying mechanisms that allow one to see what is heard.

### Conflict of interest statement

The authors declare that the research was conducted in the absence of any commercial or financial relationships that could be construed as a potential conflict of interest.

## Footnotes

^1^www.seeingwithsound.com/acuity.htm

^2^www.seeingwithsound.com

^3^www.tonometric.com
